# Excess death estimates compared with state-reported and observed COVID-19 deaths, New Jersey and the United States, 2020–2022

**DOI:** 10.3389/fpubh.2024.1338579

**Published:** 2024-08-21

**Authors:** Diana Reichbind, Lemlem Mehari, Mojisola Ojo, Nagla Bayoumi, Edward Lifshitz

**Affiliations:** ^1^Centers for Disease Control and Prevention Foundation Assignee to the Communicable Disease Service, Infectious and Zoonotic Disease Program, Division of Epidemiology, Environmental, and Occupational Health, New Jersey Department of Health, Trenton, NJ, United States; ^2^Communicable Disease Service, Infectious and Zoonotic Disease Program, Division of Epidemiology, Environmental, and Occupational Health, New Jersey Department of Health, Trenton, NJ, United States

**Keywords:** excess death estimates, COVID-19 deaths, overreporting, underreporting, New Jersey, United States

## Abstract

Deaths associated with COVID-19 in the United States are currently estimated to be over 1.2 million, but the true burden of mortality due to the SARS-CoV-2 virus is unknown. Methods for identifying and reporting deaths related to COVID-19 differ between jurisdictions, and concerns about overreporting and underreporting exist. Excess death estimates for the pandemic period, based on data from the National Center for Health Statistics, may be used to approximate the number of COVID-19-associated deaths. In this analysis, we first describe the process by which the New Jersey Department of Health identified, classified, and reported COVID-19-associated deaths from January 2020 through December 2022. The National Center for Health Statistics’ excess deaths estimates are first compared with New Jersey’s reported COVID-19-associated deaths, and then with the observed COVID-19-associated deaths in the entire United States, by month, from January 2020 through December 2022. New Jersey’s reported COVID-19-associated deaths (*n* = 35,555) accounted for (and slightly exceeded) the state’s excess deaths estimated by the National Center for Health Statistics for 2020–2022 (*n* = 30,365). However, the overall number of United States observed COVID-19 deaths for 2020–2022 (*n* = 1,094,230) for the study period did not account for all estimated excess deaths in the nation for the same period (*n* = 1,233,366). The general congruence of New Jersey’s reported COVID-19 deaths and the National Center for Health Statistics’ excess death estimates may be due in part to New Jersey’s early detailed classification system for identifying and reporting deaths associated with COVID-19, leading to more accurate COVID-19 death reporting by the state.

## Introduction

The COVID-19 pandemic has had a devastating impact on the United States (US) with over 1 million deaths attributed to the virus as of September 2023 (1). The true toll of the pandemic may be even higher, as supported by studies indicating deaths due to COVID-19 have been undercounted (2–4). The Council of State and Territorial Epidemiologists (CSTE) released a case definition for identifying and classifying COVID-19-associated deaths on December 22, 2021 (5). Prior to then, there was no standard definition for reporting COVID-19-associated deaths, and jurisdictions may have used different methods to count these deaths. Using the same criteria to count COVID-19 deaths in all US jurisdictions allows for meaningful comparisons between communities and contributes to a more accurate national picture of the true mortality burden of the pandemic.

Since COVID-19-associated deaths may have been undercounted (2–4), one way to measure the potential burden of mortality related to the COVID-19 pandemic is to estimate the number of excess deaths. Estimates of excess deaths can be calculated in a variety of ways, and both the World Health Organization (WHO) and the US Centers for Disease Control and Prevention (CDC) have developed methodologies to estimate excess mortality associated with COVID-19. WHO’s methodology continues to be developed to account for nations that have sparse or missing COVID-19-associated death data. CDC’s excess death estimates provide data for both the US overall and for individual states/jurisdictions, which were required to report COVID-19-associated deaths during the pandemic. CDC defines excess deaths as the difference between the observed number of deaths and the expected number of deaths during a specific period (6). Excess deaths can be due to all causes, including COVID-19 and other infectious and chronic diseases. In this analysis, we describe the process by which the New Jersey Department of Health (NJDOH) identified, classified, and reported COVID-19-associated deaths from January 2020 through December 2022. Additionally, National Center for Health Statistics’ (NCHS) excess death estimates for New Jersey (NJ) and the US are compared with NJ’s reported COVID-19 deaths and with US observed COVID-19 deaths, respectively, from 2020 to 2022. These comparisons can help determine how differing reporting processes may affect the congruence between COVID-19 death counts and excess death estimates. By examining COVID-19-associated deaths reported in the US, there can be a better understanding of the true health impact the pandemic had on the nation.

## Materials and methods

### Data and analysis

The study period was from January 1, 2020, to December 31, 2022. Two data sets were used: CDC’s NCHS National and State Estimates of Excess Deaths data set (6) and the CDC Weekly United States COVID-19 Cases and Deaths by State data set (7). Since the week-ending dates differ for the two data sets, all numbers provided as weekly counts in these data sets were aggregated into monthly counts for comparison purposes. The following metrics were used to compare the two data sets: NCHS excess death estimates for NJ and the US, NJ COVID-19 deaths reported to CDC, and US observed COVID-19 deaths.

The NCHS National and State Estimates of Excess Deaths data set (6) was used for all-cause excess death estimates for NJ and the US. This data was extracted from the national Electronic Death Reporting System (EDRS) on March 6, 2023. The data set provides weekly estimates of excess deaths for national, state, and other jurisdictions, by date of death. Excess death estimates were calculated as the difference between the “observed count and one of two thresholds [either the average expected count or the upper bound of the 95% prediction interval (threshold)], by week and jurisdiction” (6). Negative values, where the observed count fell below the threshold, were set to zero.

The CDC Weekly United States COVID-19 Cases and Deaths by State data set (7) provides weekly case and death counts reported by states and territories to the CDC. The data set for NJ’s reported COVID-19 deaths was extracted on August 25, 2023. National observed death counts (provided in the NCHS National and State Estimates of Excess Deaths data set) were compared with NCHS excess death estimates. Counts for US observed COVID-19 deaths were calculated by subtracting national observed death counts for ‘All-causes – except COVID-19’ from national observed death counts for ‘All-causes’ for each month in the study period. ‘All-causes – except COVID-19’ death estimates excluded deaths with an International Classification of Diseases, Tenth Edition (ICD-10) code of U07.1 as an underlying or multiple cause of death; ‘All-causes’ included all-cause mortality.

### Classifying COVID-19 cases and ascertaining COVID-19 deaths

Classifying COVID-19 deaths as confirmed or probable in NJ between 2020 and 2022 was based on meeting the criteria for COVID-19 case ascertainment as published by CSTE in position statements released in April 2020, August 2020, and August 2021 (8–10), according to which case definition was in effect when the case expired.

Confirmed COVID-19 deaths included deceased individuals with confirmatory laboratory evidence (i.e., detection of SARS-CoV-2 RNA in a clinical specimen using a molecular amplification detection test; or detection of SARS-CoV-2 RNA in a clinical or autopsy specimen using a molecular amplification test; or detection of SARS-CoV-2 RNA in a post-mortem respiratory swab or clinical specimen using a diagnostic molecular amplification test performed by a CLIA-certified provider; or detection of SARS-CoV-2 by genomic sequencing, depending on which case definition was in place at the time of death). Probable COVID-19 deaths included individuals with presumptive laboratory evidence (i.e., detection of a specific antigen in a clinical specimen or detection of a specific antibody in serum, plasma, or whole blood indicative of a new or recent infection; or detection of SARS-CoV-2 by antigen test in a respiratory specimen; or detection of SARS-CoV-2 specific antigen in a post-mortem obtained respiratory swab or clinical specimen using a diagnostic test performed by a CLIA-certified provider), depending on the case definition in effect at the time of death. Probable COVID-19 deaths also included individuals who had died without laboratory confirmation but for whom COVID-19 disease or SARS-CoV-2 was listed as a cause of death or a significant condition contributing to death on the death certificate, as well as individuals with symptoms compatible with COVID-19 and a known COVID-19 exposure who were not tested for the SARS-CoV-2 virus.

Since the surveillance definition did not take the elapsed time between illness onset or date of positive specimen collection and date of death into consideration for associating a death to COVID-19, NJDOH collaborated with the CDC to establish additional guidelines for the review of cases and deaths before categorizing them as COVID-19-associated. Thus, COVID-19 deaths were also classified as confirmed if they met the following criteria:

1. Individuals with confirmatory laboratory evidence who expired ≤30 days of a confirmatory lab test, except if a fully explanatory alternative cause of death is known that is causally unrelated to COVID-19 (such as a homicide or accident), OR
2. Individuals with confirmatory laboratory evidence who expired >30 days after confirmatory lab test AND met at least one of the following criteria:
  - COVID-19 or equivalent is mentioned on the death certificate, or
  - COVID-19 findings/symptoms (such as ARDS or pneumonia) were documented in NJ’s electronic Communicable Disease Registry and Surveillance System (CDRSS) or on the death certificate, OR there is epidemiological evidence suggesting death is otherwise temporally and causally related to COVID-19, and:
    - The individual did not experience complete recovery back to baseline state of health (per information from local health department, hospital infection preventionist, or long-term care facility), and
    - The individual did not have a fully explanatory alternative cause of death that is determined to be causally unrelated to COVID-19 (i.e., homicide, accident, etc.).

The following criteria were used in NJ to classify COVID-19 deaths as probable:

1. COVID-19 or equivalent is mentioned on the death certificate, OR
2. Individuals with presumptive laboratory evidence that met COVID-19 clinical (via CDRSS or death certificate) or epidemiological criteria (according to the surveillance case definition) who expired ≤30 days after presumptive lab test, OR
3. Individuals with presumptive laboratory evidence that met COVID-19 clinical (CDRSS or death certificate) criteria according to the surveillance case definition who expired >30 days after presumptive lab test, and:
  - The individual did not experience complete recovery back to baseline state of health (determined as described above), and
  - The individual did not have a fully explanatory alternative cause of death that is determined to be causally unrelated to COVID-19 (such as a homicide or accident), OR
4. Individuals who met COVID-19 clinical (CDRSS or death certificate) and epidemiological criteria according to the surveillance case definition (no positive COVID-19 test results, meaning either no COVID-19 testing was performed or negative test results were present but preceded the individual’s terminal illness onset as determined by CDRSS record review) who expired ≤30 days of symptom onset, OR
5. Individuals who met COVID-19 clinical (CDRSS or death certificate) and epidemiological criteria according to the surveillance case definition (no positive COVID-19 test results, as defined above) who expired >30 days of symptom onset, and.
  - The individual did not experience complete recovery back to baseline state of health, and
  - The individual did not have a fully explanatory alternative cause of death that is determined to be causally unrelated to COVID-19 (i.e., accident, homicide).

Between 2020 and 2022, NJDOH used two sources to quantify deaths associated with COVID-19: Electronic death records data and public health disease surveillance and investigation. Electronic death records data from the New Jersey Electronic Death Records System (NJ-EDRS) were pulled and matched to COVID-19 cases in CDRSS using demographic data manually reviewed by NJDOH staff 5–7 days a week. Information on matched cases was utilized to update the mortality status of known COVID-19 cases whose deaths were not reported by the LHD, to verify COVID-19 as the cause of death on known fatalities of COVID-19 cases, and to identify COVID-19 cases that were not reported through public health reporting and surveillance. The majority of COVID-19-associated deaths reported by NJ were identified through this mechanism, which minimized lag time between the date of death and the reporting date as NJDOH had access to NJ-EDRS data in real-time. This also decreased the workload on local health departments (LHD) to identify and report COVID-19-associated deaths to NJDOH. LHDs received reports of COVID-19-associated deaths from healthcare facilities and local death registrars. Additionally, they identified confirmed and probable deaths through investigations of persons who tested positive for COVID-19, were symptomatic close contacts of a COVID-19 case, or were associated with a COVID-19 outbreak or cluster.

## Results

**Figure 1:**
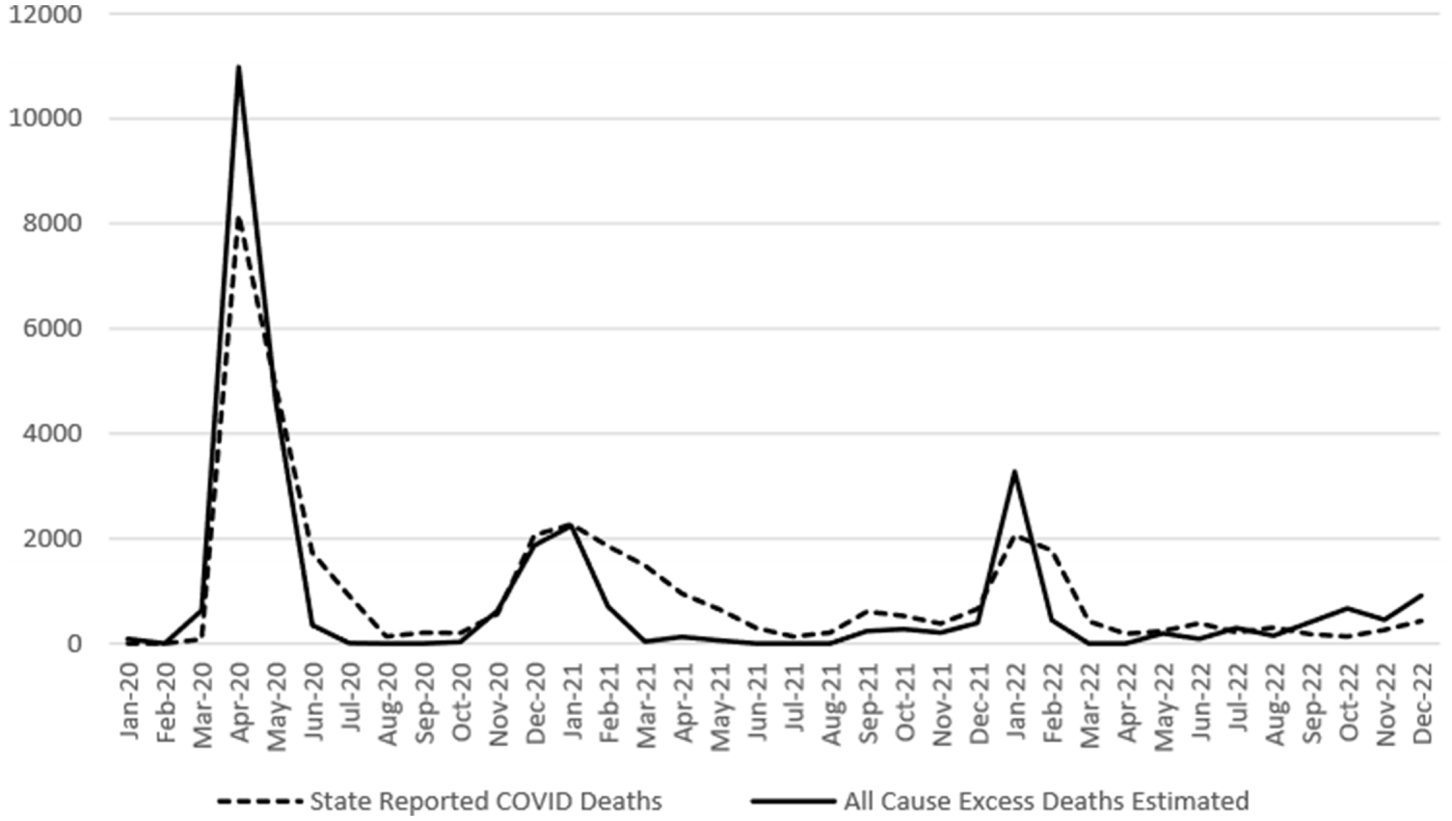
NJ Reported COVID-19 deaths compared with NCHS excess death estimates, 2020–2022. Sources: CDC COVID-19 provisional counts weekly death estimates (6), CDC weekly United States COVID-19 cases and death by state (7).

NJDOH began reporting deaths associated with COVID-19 in March 2020. As shown in Figure 1, NJ’s reported COVID-19 deaths closely track NCHS excess death estimates for the state, although reporting in some months did exceed or fall below excess death estimates due to lag times in death certificate reporting, incomplete medical histories, and human error. NJ-reported COVID-19 deaths exceeded estimated excess deaths the most in June and July 2020, from February through May 2021, and in February 2022 (Table 1). NJ reported substantially fewer COVID-19 deaths than excess death estimates in March and April 2020, in January 2022, and in October and December 2022. Overall, NJ-reported COVID-19-associated death counts (*n* = 35,555) ultimately accounted for and slightly exceeded the total excess deaths estimated by NCHS for NJ for 2020–2022 (*n* = 30,365).

**Table 1:** Differences between NCHS all-cause excess death estimates (NJ and US) and reported (NJ)/observed (US) COVID-19 deaths by month, January 2020 – December 2022.

Month/year	Estimated NJ all-cause excess deaths	NJ reported COVID deaths	Difference (NJ estimated - NJ reported)	Estimated US all-cause excess deaths	US observed COVID deaths	Difference (US estimated - US observed)
Jan-2020	90	0	90	0	5	−5
Feb-2020	0	0	0	0	21	−21
Mar-2020	630	81	549	3,393	3,901	−508
Apr-2020	10,984	8,173	2,811	70,108	59,207	10,901
May-2020	4,623	4,889	−266	43,720	47,107	−3,387
Jun-2020	346	1,729	−1,383	16,518	16,985	−467
Jul-2020	10	906	−896	34,570	25,780	8,790
Aug-2020	1	136	−135	46,062	35,577	10,485
Sep-2020	0	208	−208	22,724	18,226	4,498
Oct-2020	26	202	−176	30,702	27,285	3,417
Nov-2020	621	562	59	53,248	48,398	4,850
Dec-2020	1,874	2,066	−192	89,289	85,203	4,086
Jan-2021	2,238	2,268	−30	115,910	120,742	−4,832
Feb-2021	703	1,857	−1,154	29,868	50,125	−20,257
Mar-2021	37	1,484	−1,447	1,066	21,850	−20,784
Apr-2021	125	947	−822	809	17,613	−16,804
May-2021	60	651	−591	3,936	17,917	−13,981
Jun-2021	0	297	−297	4,925	7,823	−2,898
Jul-2021	0	131	−131	14,691	12,089	2,602
Aug-2021	1	208	−207	53,423	42,337	11,086
Sep-2021	235	605	−370	72,45	60,602	11,848
Oct-2021	274	529	−255	61,489	50,886	10,603
Nov-2021	211	379	−168	40,349	29,699	10,650
Dec-2021	391	659	−268	51,294	39,278	12,016
Jan-2022	3,277	2,062	1,215	81,807	88,338	−6,531
Feb-2022	442	1,771	−1,329	74,599	53,942	20,657
Mar-2022	0	425	−425	8,103	16,076	−7,973
Apr-2022	0	186	−186	2,105	7,643	−5,538
May-2022	193	240	−47	11,001	6,786	4,215
Jun-2022	91	385	−294	16,749	8,659	8,090
Jul-2022	293	214	79	28,084	14,544	13,540
Aug-2022	151	301	−150	24,020	12,828	11,192
Sep-2022	407	181	226	23,493	10,789	12,704
Oct-2022	664	135	529	29,782	11,149	18,633
Nov-2022	455	257	198	25,948	9,166	16,782
Dec-2022	912	431	481	47,131	15,654	31,477
Total	30,365	35,555	−5,190	1,233,366	1,094,230	+139,136

**Figure 2:**
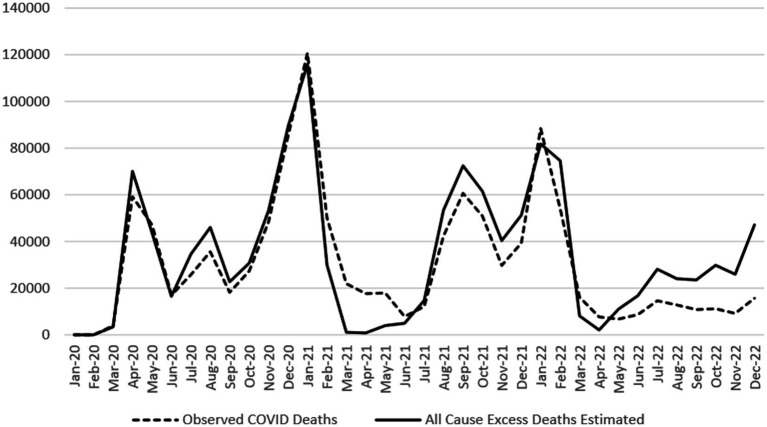
US observed COVID-19 deaths compared with NCHS excess death estimates, 2020–2022. Source: CDC COVID-19 provisional counts weekly excess deaths by Jurisdiction (6).

Observed COVID-19 deaths in the US are compared with NCHS excess death estimates for the US in Figure 2. Observed deaths exceeded excess death estimates the greatest from February through May 2021, in January 2022, and in March and April 2022 (Table 1). Observed US deaths were markedly lower than estimated deaths for several months earlier in the pandemic (April, July and August 2020), as well as later in 2021 (August through December), and in much of 2022 (February and June through December). Decreased congruity is seen with this comparison, with larger numbers of excess estimated deaths than observed COVID-19 deaths for close to half the months in the study period. The overall number of US-observed COVID-19 deaths from January 2020 through December 2022 (*n* = 1,094,230) falls short of accounting for all estimated excess deaths in the US for the same period (*n* = 1,233,366) by over 11%.

## Discussion

Overall, NJ’s reported COVID-19 deaths from 2020 to 2022 closely align with excess death estimates produced by NCHS and account for all estimated excess deaths during that period. This is consistent with the absence of any other historical events causing widespread fatalities in either NJ or the US at that time. The general congruence of NJ-reported COVID-19 deaths and NCHS excess death estimates may be partly the result of establishing an early detailed classification system for identifying, counting and reporting deaths associated with COVID-19, leading to accurate COVID-19 death reporting in NJ. The inclusion of criteria for classifying both confirmed and probable COVID-19 deaths in NJ, as well as the presence of two mechanisms by which deaths associated with COVID-19 were identified by NJDOH (public health investigation/disease surveillance and electronic death records/death certificate data), has established a robust system that identified and reported COVID-19-associated deaths accurately overall. Despite the general agreement between the NJ-reported COVID-19 deaths and the NCHS excess death estimates, NJ-reported COVID-19-associated deaths for the study period exceeded overall NJ excess death estimates by approximately 17%. This difference may at least partially due to the continued high levels of circulating COVID-19 during the study period in NJ, which could have resulted in higher COVID-19 deaths than provisional excess death estimates had accounted for. Additionally, the number of NJ-reported deaths associated with COVID-19 may be a more accurate reflection of the true mortality burden of the pandemic in the state, given the indirect effects on health for the public (such as deferred illness screenings and treatments during hospital surges, increases in depression and social isolation, and elevated abuse of substances, all of which negatively affect health and contribute to higher mortality). Unlike in NJ, the total number of observed COVID-19 deaths in the US did not account for all the estimated excess deaths for the same period. This suggests a potential underreporting of COVID-19 deaths across the US.

Limitations include the possibility that the weighting method used in excess death estimates could over-adjust for underreporting, thereby producing inflated excess death estimates (6). However, given the lack of accessible and reliable testing for COVID-19 at the beginning of the pandemic (therefore artificially decreasing deaths associated with COVID-19 since no testing was performed), the issue that decedents may not have been tested for COVID-19 even after testing became more widely available (and so may not have had a COVID-19 diagnosis listed as a cause of death on their death certificates), and the differing methods and criteria used to identify, classify and count COVID-19-associated deaths, underreporting of these deaths is a distinct possibility for many jurisdictions. Differing methods can lead to different results; therefore, jurisdictional comparisons of COVID-19 deaths should be approached with great caution. Additionally, because the excess death estimates used in this study were for all-cause mortality, some of the pandemic’s secondary effects on mental health, substance abuse patterns, healthcare services, etc. are likely captured in these estimates. However, since many of these effects have not been quantified and measured, it would not be possible to determine the extent to which they influenced trends in mortality in this study. Finally, since aggregated US data were not available in the CDC Weekly United States COVID-19 Cases and Deaths by State data set, the category ‘US observed COVID-19 deaths’ was calculated as a proxy to compare with NCHS excess death estimates for the US jurisdiction, and a direct comparison could not be performed. Comparisons between excess death estimates and reported/observed deaths by age group and gender also were not performed, as these breakdowns were not included in the data sets.

There continues to be debate about the underreporting and/or overreporting of COVID-19-associated deaths (11, 12). Despite this, NCHS excess death estimates are available to help validate the numbers of jurisdiction-reported COVID-19 deaths. These estimates are used by the public health community and serve as the standard by which many jurisdictions calculate excess deaths caused by COVID-19 during the pandemic. NJDOH established a process for identifying, classifying and reporting COVID-19-related deaths early in the pandemic. The process included reporting both confirmed and probable deaths; this approach seems to have allowed for NJ’s reported COVID-19 deaths to track similarly to NCHS excess death estimates throughout most of the pandemic, and ultimately account for the jurisdiction’s estimated excess deaths from 2020 to 2022. This may be a useful approach for other jurisdictions where excess death estimates exceed reported COVID-19 deaths to more accurately understand the true burden of mortality caused by the pandemic. Accurate COVID-19 death reporting allows for understanding the human toll of the pandemic and targeting resources to jurisdictions with the greatest need. Additionally, improving death reporting and registration systems would allow for improved monitoring not just for COVID-19, but for any future pandemics as well.

## Data Availability

Publicly available datasets were analyzed in this study. This data can be found at: https://covid.cdc.gov/covid-data-tracker/#datatracker-home, https://data.cdc.gov/Case-Surveillance/Weekly-United-States-COVID-19-Cases-and-Deaths-by-/pwn4-m3yp.
